# Senescence-related genes define prognosis, immune contexture, and pharmacological response in gastric cancer

**DOI:** 10.18632/aging.204524

**Published:** 2023-02-16

**Authors:** Xiaogang Shen, Meng Wang, Wenxi Chen, Yu Xu, Qiaoxia Zhou, Tengfei Zhu, Guoqiang Wang, Shangli Cai, Yusheng Han, Chunwei Xu, Wenxian Wang, Lei Meng, Hao Sun

**Affiliations:** 1 Departments of gastrointestinal surgery, Sichuan Academy of Medical Sciences and Sichuan Provincial People’s Hospital, Chengdu, China; 2 Department of General Surgery, The Third People’s Hospital of Chengdu, Affiliated Hospital of Southwest Jiaotong University, Chengdu, China; 3 Burning Rock Biotech, Guangzhou, China; 4 Institute of Basic Medicine and Cancer (IBMC), Chinese Academy of Sciences, Hangzhou, China; 5 Department of Clinical Trial, The Cancer Hospital of the University of Chinese Academy of Sciences (Zhejiang Cancer Hospital), Hangzhou, China; 6 Department of Surgical Oncology, The First Affiliated Hospital of Xi'an Jiaotong University, Xi’an, China; 7 Department of Gastrointestinal Cancer Center, Chongqing University Cancer Hospital, Chongqing, China

**Keywords:** gastric cancer, senescence, prognosis, tumor immune microenvironment

## Abstract

As one of the prevalent tumors worldwide, gastric cancer (GC) has obtained sufficient attention in its clinical management and prognostic stratification. Senescence-related genes are involved in the tumorigenesis and progression of GC. A machine learning algorithm-based prognostic signature was developed from six senescence-related genes including *SERPINE1*, *FEN1*, *PDGFRB*, *SNCG*, *TCF3*, and *APOC3*. The TCGA-STAD cohort was utilized as a training set while the GSE84437 and GSE13861 cohorts were analyzed for validation. Immune cell infiltration and immunotherapy efficacy were investigated in the PRJEB25780 cohort. Data from the genomics of drug sensitivity in cancer (GDSC) database revealed pharmacological response. The GSE13861 and GSE54129 cohorts, single-cell dataset GSE134520, and The Human Protein Atlas (THPA) database were utilized for localization of the key senescence-related genes. Association of a higher risk-score with worse overall survival (OS) was identified in the training cohort (TCGA-STAD, P<0.001; HR = 2.03, 95% CI, 1.45–2.84) and the validation cohorts (GSE84437, *P* = 0.005; HR = 1.48, 95% CI, 1.16–1.95; GSE13861, *P* = 0.03; HR = 2.23, 95% CI, 1.07–4.62). The risk-score was positively correlated with densities of tumor-infiltrating immunosuppressive cells (*P* < 0.05) and was lower in patients who responded to pembrolizumab monotherapy (*P* = 0.03). Besides, patients with a high risk-score had higher sensitivities to the inhibitors against the PI3K-mTOR and angiogenesis (*P* < 0.05). Expression analysis verified the promoting roles of *FEN1*, *PDGFRB*, *SERPINE1*, and *TCF3*, and the suppressing roles of *APOC3* and *SNCG* in GC, respectively. Immunohistochemistry staining and single-cell analysis revealed their location and potential origins. Taken together, the senescence gene-based model may potentially change the management of GC by enabling risk stratification and predicting response to systemic therapy.

## INTRODUCTION

Gastric cancer is one of the most prevalent tumors, with the fifth-highest incidence and fourth-highest mortality rate all over the world [1]. Exploring prognostic and therapeutic biomarkers in GC is of great importance and urgency. Cancer is an aging disease and cellular senescence plays an essential role in promoting cancer development and tumor progression [2], suggesting the great potential of senescence-related genes in predicting prognosis and pharmacological response.

In mammalian cells, stimulated oncogenes accompanied by inactivated tumor-suppressor genes (TSGs) are crucial inducements of proliferative stress and induction of cellular senescence, which therefore limit tumor growth [3–5]. For instance, expression of *HRAS^G12V^* is usually associated with upregulated senescence-related genes including *p53*, *p19^ARF^*, *p16^INK4a^, Pml*, and retinoblastoma, which work as an obstructive factor for tumor initiation [6, 7]. However, further stimulation of oncogenes or deactivation of TSGs elicits bypass of the previous senescence, contributing to tumorigenesis [8, 9].

Senescence-related secretory phenotype (SASP) refers to the ability of senescent tumor cells to actively produce a wide variety of proteins, many of which are pro-inflammatory cytokines or pro-inflammatory substances in themselves [10, 11]. SASP is a double-edged sword due to its both antitumorigenic and cancer-promoting impact by propagating senescence to other tumor cells and recruiting immune cells to clear senescence tumor cells, respectively [12–15]. Given the regulatory effect of tumoral senescence on tumor-infiltrating immune cells, we hypothesized that the activation of senescence-related genes may be involved in immune cell infiltration and thereby affect immunotherapy efficacy in GC.

Here, based on senescence-related genes, we sought to develop a model for the prognostic stratification of GC. A favorable prognosis was observed in the low-risk group, together with low sensitivities to the inhibitors against the PI3K-mTOR and angiogenesis, low densities of immunosuppressive tumor-infiltrating immune cells, and a high response rate to pembrolizumab monotherapy.

## RESULTS

### Analysis of differentially expressed genes for potential prognostic signature

**Figure 1:**
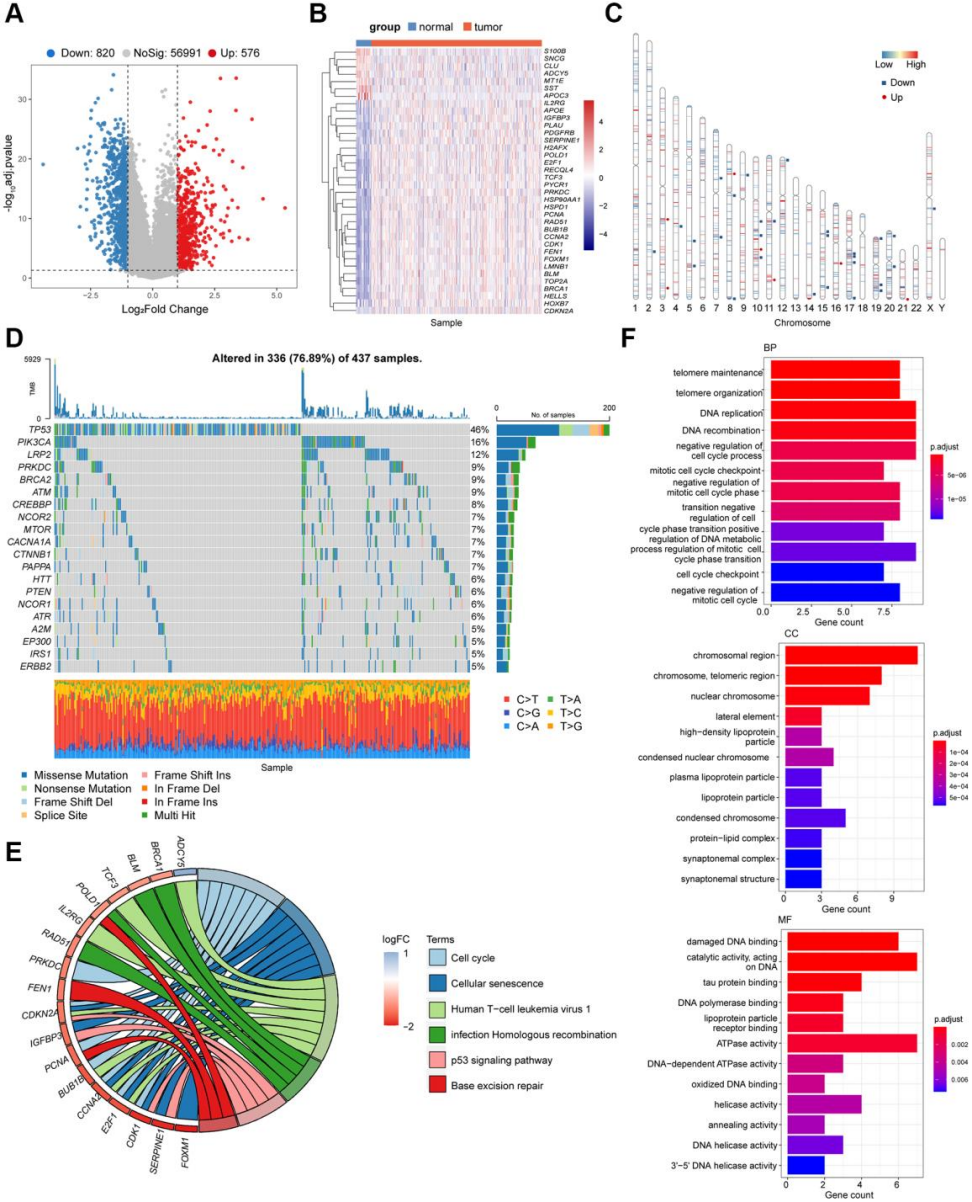
**Identification of the candidate senescence-related DEGs in the TCGA-STAD.** (**A**) Differentially expressed genes depicted by the volcano plot (red, up-regulated; blue, down-regulated in GC). (**B**) Heatmap depicting the mRNA levels of the 36 senescence-related DEGs between GC tissues and adjacent normal tissues. (**C**) Locations of the 36 senescence-related DEGs in chromosomes (red, up-regulated; blue, down-regulated in GC). (**D**) The mutation frequency of top 20 DEGs. (**E**) Bubble diagram demonstrated the top 6 enriched KEGG pathways of the 36 senescence-related DEGs. (**F**) GO enrichment analysis of the 36 senescence-related DEGs via biological process (BP), cellular component (CC) and molecular function (MF).

Baseline characteristics of the patients used in the training and validation sets were depicted in Supplementary Table 1. We first tried to identify senescence-related differentially expressed genes (DEGs) in patients with GC. In total, 1,396 DEGs between tumor and non-tumorous tissues in the cancer genome atlas-stomach adenocarcinoma (TCGA-STAD) cohort were identified (Figure 1A). Of these, 36 genes were senescence-related genes (Figure 1B). The chromosomal locations of these senescence-related DEGs are shown in Figure 1C. We also demonstrated the mutations in the 36 senescence-related DEGs in GC patients and the top 20 most mutated senescence-related DEGs in Figure 1D. The mutational frequency of *TP53* was the highest (46%) followed by *PIK3CA* (16%, Figure 1D).

According to the Kyoto Encyclopedia of Genes and Genomes (KEGG) pathway analysis, these DEGs were mainly enriched in cell cycle regulation, homologous recombination, base excision repair, and P53 pathway (*P* < 0.05, Figure 1E). As expected, the 36 senescence-related DEGs were involved in DNA replication, telomere maintenance, negative cell cycle regulation, and DNA metabolism (*P* < 0.05, Figure 1F), which are consist in pathways related to cell cycle and cellular senescence. These findings collectively suggested the potential association between the senescence-related DEGs and the tumorigenesis of GC.

### Prognostic model construction and validation

**Figure 2:**
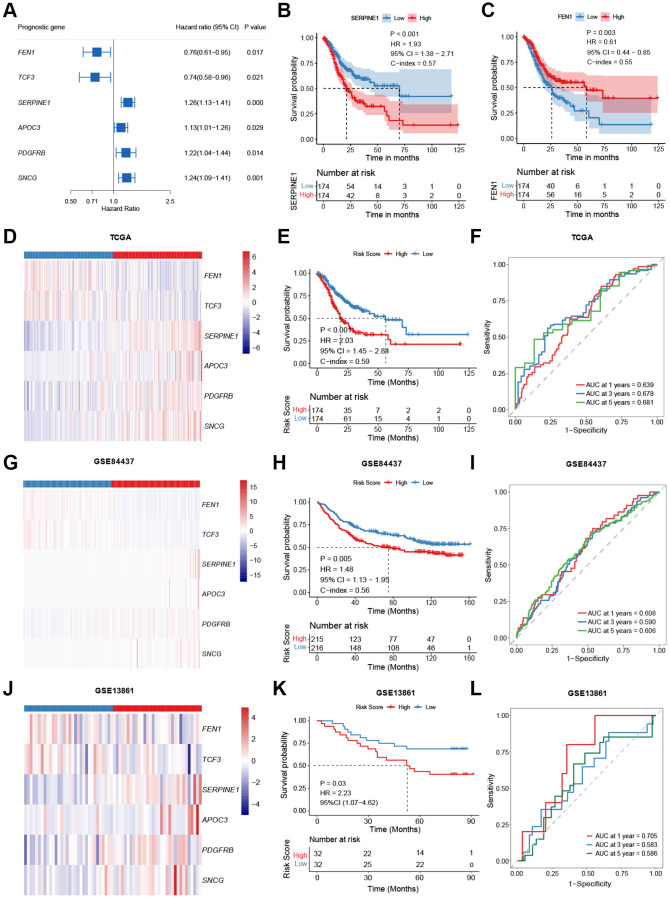
**Model construction and validation.** (**A**) Potential prognostic valued of each senescence-related genes in the overall survival (OS) of gastric cancer (GC). (**B**, **C**) Kaplan-Meier curves comparing the OS between patients with high and low expressions of *SERPINE1* (**B**) and *FEN1* (**C**), respectively. (**D**–**L**) Heatmap, Kaplan-Meier curves, and ROC curves depicting the gene expression patterns, survival status, and prognostic valued of the model in the TCGA-STAD (**D**–**F**), the GSE84437 (**G**–**I**), and the GSE13861 (**J**–**L**), respectively.

Of the 36 senescence-related DEGs, six senescence-related DEGs were identified due to their association with overall survival (OS) as continuous variables in the TCGA-STAD cohort (*P* < 0.05, Figure 2A, Supplementary Table 2). For instance, poorer OS was observed in patients with higher expression of *SERPINE1* (*P* < 0.001; hazard ratio (HR) = 1.93; 95% confidence interval (95% CI), 1.38–2.71; Figure 2B), while patients with high expression of *FEN1* exhibited improved OS (*P* = 0.003;HR, 0.61; 95% CI, 0.44–0.85; Figure 2C).

Based on the mRNA levels of these six genes, a risk-score was then developed and defined as follows: risk-score = (0.196 × *SERPINE1*) + (0.120 × *APOC3*) + (0.090 × *SNCG*) + (0.015 × *PDGFRB*) – (0.128 × *TCF3*) – (0.133 × *FEN1*). Assigned with a risk-score, patients were stratified into high- or low-risk groups by the median value in the cohort. Patients in the high-risk group had higher expression of *SERPINE1*, *APOC3*, *PDGFRB*, and *SNCG* and lower expression of *FEN1* and *TCF3* (*P* < 0.001, Figure 2D). In the TCGA-STAD cohort, the low-risk group exhibited improved OS (*P* < 0.001; HR = 2.03; 95% CI, 1.45–2.84; Figure 2E). The 1-, 3-, and 5-year area under curves (AUCs) of the risk-score were 0.639, 0.678, and 0.681, respectively (Figure 2F). These results were further verified in two validation cohorts (GSE84437 and GSE13861). Patients with higher risk had higher levels of *SERPINE1*, *APOC3*, *PDGFRB,* and *SNCG,* and lower *FEN1* and *TCF3* expressions (GSE84437: Figure 2G, GSE13861: Figure 2J, *P* < 0.01), together with worse OS (GSE84437: *P* = 0.005; HR = 1.48, 95% CI, 1.13–1.95; Figure 2H; GSE13861: *P* = 0.03; HR = 2.23, 95% CI, 1.07–4.62; Figure 2K). The signature predicted 1-, 3-, and 5-year OS with AUCs of 0.608, 0.590, and 0.606 in the GSE84437 cohort, and 0.705, 0.583 (Figure 2I), and 0.586 in the GSE13861 cohort (Figure 2L), respectively.

Univariable and multivariable Cox regression analysis was conducted to examine the independence of the novel prognostic signature. After adjusted for key covariates including TNM stage and age, the signature remained robust in OS differentiation in the TCGA-STAD cohort (*P* < 0.001; HR = 2.23, 95% CI, 1.57–3.12; Table 1), the GSE84437 cohort (*P* = 0.02; HR = 1.40, 95% CI, 1.07–1.85; Table 1), and the GSE13861 cohort (*P* = 0.10; HR = 1.87, 95% CI, 0.87–4.03; Table 1). The results concerning the independence of the six-gene signature were consistent between the three cohorts, indicating the robustness of our model in predicting prognosis.

**Table 1:** Univariable and multivariable Cox regression in TCGA-STAD and GSE84437 cohorts.

**Parameter**	**Univariable analysis**		**Multivariable analysis**	
	**HR (95% CI)**	***P* value**	**HR (95% CI)**	***P* value**
**TCGA-STAD cohort**				
Age (≥65 vs. <65)	1.49 (1.06–2.10)	0.02	1.67 (1.17–2.37)	0.01
Sex (male vs. female)	1.35 (0.95–1.94)	0.10		
Tumor stage (I and II vs. III and IV)	1.65 (1.09–2.49)	0.02	1.78 (1.16–2.74)	0.01
EBV infection (positive vs. negative)	0.94 (0.48–1.85)	0.86		
MSI (MSI-H vs. MSI-L and MSS)	1.94 (0.53–7.11)	0.19		
*TP53* (mutation vs. wildtype)	0.65 (0.32–0.84)	0.06		
Asian (yes vs. no)	0.59 (0.37–0.95)	0.03	0.54 (0.33–0.87)	0.01
*SMARCA4* (mutation vs. wildtype)	0.45 (0.16–1.28)	0.13		
**Risk score (high-risk vs. low-risk)**	**2.03 (1.45–2.84)**	**<0.001**	**2.23 (1.57–3.12)**	**<0.001**
**GSE84437 cohort**				
Age (≥65 vs. <65)	1.37 (1.04–1.81)	0.02	0.73 (0.56–0.97)	0.03
Sex (male vs. female)	1.24 (0.91–1.77)	0.17		
Tumor stage (I and II vs. III and IV)	3.71 (1.90–7.24)	<0.001	0.28 (0.14–0.54)	<0.001
**Risk score (high-risk vs. low-risk)**	**1.48 (1.13–1.95)**	**0.005**	**1.40 (1.07–1.85)**	**0.02**
**GSE13861 cohort**				
Age (≥65 vs. <65)	1.20 (0.58–2.52)	0.62		
Sex (male vs. female)	1.27 (0.59–2.73)	0.55		
Tumor stage (I and II vs. III and IV)	7.70 (2.32–25.54)	<0.001	7.12 (2.14–23.70)	<0.001
**Risk score (high-risk vs. low-risk)**	**2.24 (1.04–4.83)**	**0.04**	**1.87 (0.87–4.03)**	**0.1**

Abbreviations: EBV: Epstein-Barr virus; MSI: microsatellite instability; MSS: microsatellite stability.

### Role of the senescence-related risk-score in mutational events, immunoinfiltration, and response to systemic therapy

**Figure 3:**
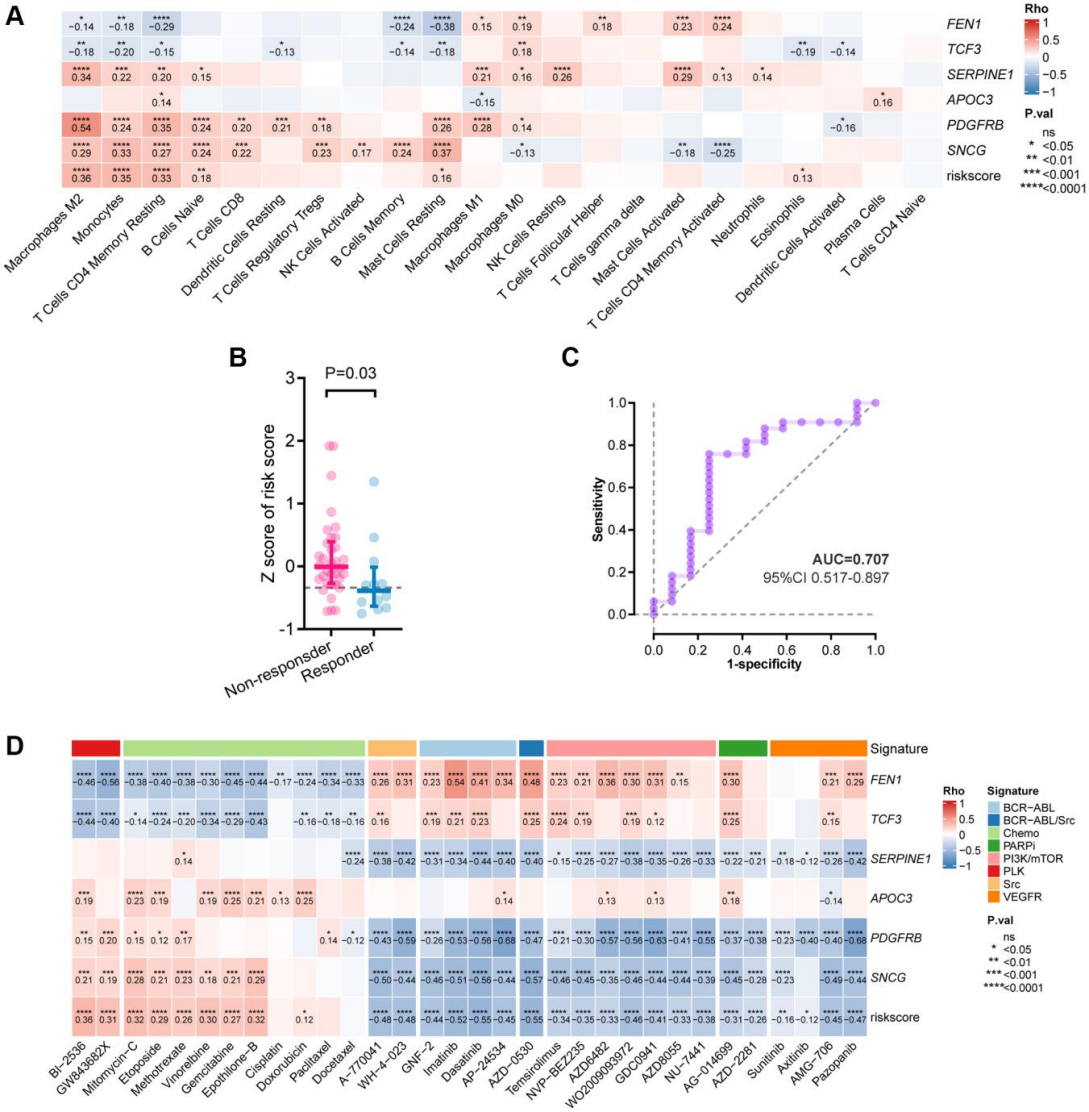
**Correlation between the six-gene signature and tumor immune features.** (**A**) Correlation between immune infiltration and the risk-score by Spearman analysis. (**B**) Comparison of the risk-score between responders and non-responders to immunotherapy in PRJEB25780 cohort. (**C**) Time-dependent ROC curve for the risk-score in predicting response to immunotherapy (**D**) Heatmap showing the Spearman analysis results of the risk-score in drug sensitivity.

We further explored the underlying difference between risk groups based on the senescence-related DEGs. Higher-risk patients had fewer mutations in *LRP1B*, *SYNE1*, and *ARID1A* (Supplementary Figure 1A, 1B), while those with lower risk-scores obtained a increased tumor mutational burden (TMB) (*P* < 0.001, Supplementary Figure 1C), suggesting that the low-risk group might be immune-sensitive since a high TMB might be linked to an inflammatory tumor immune microenvironment (TIME) and preferable sensitivity to immune checkpoint inhibitors (ICIs) [16]. Thus, we further studied the correlations of the absolute densities of 22 types of immune cells with the signature. Positive correlation was identified between the risk-score and the infiltration levels of the immune cells related to an immunosuppressive microenvironment (Figure 3A), e.g., M2 macrophage (Rho = 0.36, *P* < 0.001), resting memory CD4^+^ T cell (Rho = 0.33, *P* < 0.001), naïve B cells (Rho = 0.18, *P* = 0.003), and resting mast cells (Rho = 0.16, *P* = 0.01). Consistent with the risk-score, expression of *SERPINE1*, *PDGFRB,* and *SNCG* were also positively associated with M2 macrophage, resting memory CD4^+^ T cell, and naïve B cells, while expression of *FEN1* and *TCF3* were negatively associated with M2 macrophage, resting memory CD4^+^ T cell, and resting mast cells (Figure 3A).

Given this, we further investigated the potential of the risk-score in predicting response to ICIs based on a cohort of patients with advanced GC treated with pembrolizumab monotherapy. Patients who responded to pembrolizumab had a lower risk-score than those who didn’t (*P* = 0.03, Figure 3B), and an association between a low risk-score and objective response was observed (AUC = 0.707, 95% CI, 0.517–0.897; Figure 3C).

Besides ICIs, we examined the correlations between the risk-score and the anti-tumor efficacy of multiple treatments in GC cell lines (Figure 3D). As for chemotherapeutic drugs, a high risk-score was correlated with decreased sensitivities to gemcitabine, doxorubicin, and etoposide, etc. As for targeted agents, the correlations were observed between a high risk-score and increased sensitivities to the inhibitors targeting phosphatidylinositol 3-kinase /mammalian target of rapamycin (PI3K/mTOR), poly ADP-ribose polymerase (PARP), Polo-like kinase (PLK), Src, and vascular endothelial growth factor receptor (VEGFR). Taken together, these results may indicate the role of the risk-score in predicting response to systemic therapy in GC.

### Expression of the genes involved in the senescence-related risk-score

**Figure 4:**
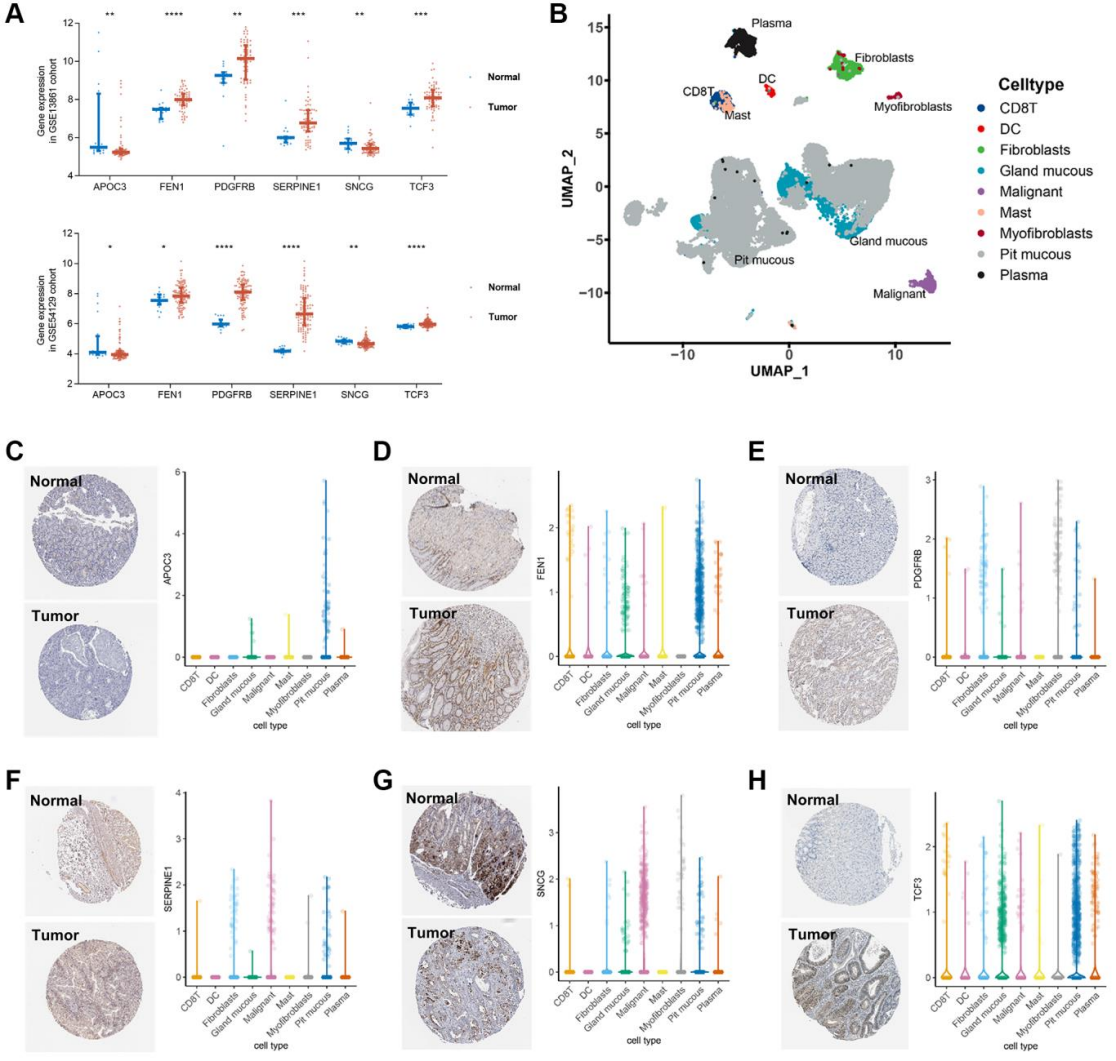
**Validation of the key genes expression in GC tissue and single cell localization.** (**A**) Expression of SERPINE1, FEN1, PDGFRB, SNCG, TCF3, and APOC3 between GC tumor and normal tissues in the GSE54129 cohort. (**B**) Seven cell types identified in the gastric cancer GSE134520 dataset by single cell RNA sequencing (scRNA-seq) profiles and the calculation of uniform manifold approximation and projection (UMAP). (**C**–**H**) Immunohistochemistry staining in stomach normal tissues (left, upper) and gastric tumor tissues (left, lower), and scRNA-seq expression levels of APOC3 (**C**), FEN1 (**D**), PDGFRB (**E**), SERPINE1 (**F**), SNCG (**G**), and TCF3 (**H**), respectively.

To further explore the potential roles of the key genes involved in the senescence-related risk-score in the tumorigenesis and development of GC by comparing the expression of *SERPINE1*, *FEN1*, *PDGFRB*, *SNCG*, *TCF3*, and *APOC3* between tumor and normal tissues. Based on the mRNA expression profile of GC tumor and adjacent normal tissues from the GSE13861 and GSE54129 cohorts, *FEN1*, *PDGFRB*, *SERPINE1*, and *TCF3* were up-regulated in tumor tissues (Figure 4A), which coincide with their risk roles in the senescence-related signature. In contrast, the lower expression levels of *APOC3* and *SNCG* in tumor (Figure 4A), together with their protective roles in the prognostic signature, have further revealed their potential suppressor functions in GC.

Besides, the data of single-cell sequencing and immunohistochemistry (IHC) staining revealed the distribution of the 6 senescence-based risk-score-related genes in GC. According to the single-cell RNA sequencing profile of the GSE134520 dataset, a total of 9 cell types were identified (Figure 4B). *APOC3* was mainly expressed in pit mucous cells, while *SNCG* expression was enriched in myofibroblasts and tumor cells. The expressions of *FEN1* and *TCF3* were mainly enriched in pit mucous and grand mucous cells, while *SERPINE1* was mainly expressed in tumor cells. Expression of *PDGFRB* was comparable in almost all cell types except mast cells. (Figure 4C–4H).

## DISCUSSION

Studies about GC, one of the most prevalent gastrointestinal (GI) malignancies, have increasingly concentrated on the prognostic implications of several signatures [17, 18]. Based on the senescence-related DEGs, a novel signature was constructed herein, which can realize patient stratification for the prognosis of GC. An improved OS was observed in patients with low-risk scores. In addition, the high-risk group exhibited a higher abundance of immunosuppressive cells, suggesting that they might benefit from ICIs. Indeed, risk-scores were lower in patients who responded to immunotherapy compared with those who did not respond in the PRJEB25780 cohort. Altogether, we developed a six-senescence-gene prognostic model, which can not only differentiate the prognosis but also guide potential treatment.

In earlier research, prognostic models for GC patients were developed using sequencing data and clinicopathologic indicators [19–22]. Clinicopathologic features such as the tumor stage, histologic grade, abnormal tumor markers, and lymphovascular space invasion are widely used to evaluate the prognosis of GC patients [23]. Utilizing gene expression patterns of GC patients from the TCGA and GEO databases, we were able to find a trustworthy indicator of GC prognosis. Our prognostic signature is of great potential to be easily applied to clinical practice for individualized prediction of GC survival. In addition, our research has another advantage. The six DEGs offer a promising assay, which is practical in actual clinical settings due to a low cost, short turn-around time, and no reliance on bioinformatics expertise. Reverse transcription-polymerase chain reaction (RT-PCR) can be easily implemented in the clinical setting, making it attractive for an easier clinical translation. The six DEGs observed in our study were of significant prognostic value, allowing the risk stratification of GC patients.

The biological features of GC may aid in predicting which tumors will benefit from chemotherapy and other targeted agents [24]. Compared to the traditional prognostic models, our model can provide additional biological features, such as TIME. Evidence in recent years has repeatedly highlighted that the interactions between cancer cells and TIME affect tumorigenesis [25, 26]. Prognostic signatures related to the TIME possess the considerable prospect to explore innovative molecular targets for immunological therapy and contribute to personalized patient care. Generally, the immune response is one of the most important results of cellular senescence [27, 28], which induces the enrichment of immune cells and promotes tumor growth [29]. The regulation of the key senescence-related genes in TIME, however, is largely unknown in GC. In this study, the high-risk group exhibited more intensive infiltration of M2 macrophages and worse prognosis, which coincides with two earlier studies revealing the role of M2 macrophages in tumor malignant features including migration and invasion [30, 31]. Another previous study revealed that the activated and resting T cells CD4 memory were enriched in head and neck cancer samples with high- and low-TMB, respectively [32], which is consistent with our results. Additionally, of the 6 genes involved in the signature, most were important for the chemotaxis of leukocytes, angiogenesis of tumor tissue, and systematic immunological functions [33–36].

Further, the drug sensitivity analyses add evidence for our model’s association with cancer and its potential clinical application. The PI3K-mTOR signaling pathway plays an important role in cancers and its inhibitors have shown efficacy in clinical trials [37]. The PI3K-mTOR inhibitors enhance nab-paclitaxel antitumor response in GC [38]. Our model based on the six DEGs can be used for risk stratification in GC. Furthermore, it may guide the clinical application of PI3K-mTOR inhibitors. Besides, there is not much evidence to support the use of pembrolizumab in individuals with untreated GC who might not benefit from chemotherapy [39]. Given this, our study demonstrated the utility of the six-DEG signature as a model to identify the GC patients who may benefit from pembrolizumab. The associations between the risk-score and immune landscape highlight the need to further understand the mechanisms of these DEGs for the development of treatment strategies.

As for limitations, the retrospective nature of this study has determined the limited capacity of the model, and prospective validation in well-designed cohorts is required to demonstrate its clinical value. Despite the consistent results among the survival analysis of TCGA and GEO cohorts, gene expression levels, IHC staining, and single-cell sequencing results, *in vitro* and *in vivo* experimental validation were highly recommended to examine the significance of the risk-score in GC and other cancers. Besides, further promising studies are recommended to explore the linkage between the 6 senescence-related genes and response to chemotherapeutic and targeted chemicals in animal models.

In summary, a novel and robust prognostic model consisting of six senescence-related genes was developed and validated in patients with GC. Additionally, the score of this model was associated with TIME and responses to chemotherapeutic, targeted, and immunotherapeutic therapies. The senescence gene-based model can potentially change the management of GC by enabling risk stratification and predicting response to systemic therapy.

## MATERIALS AND METHODS

### Data and study design

**Figure 5:**
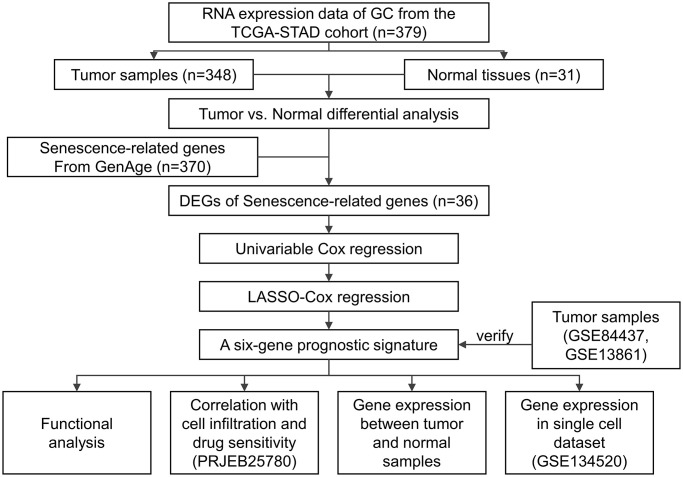
Study design.

The transcriptomic, genomic, and survival data of 348 GC and 31 controls were retrieved from the TCGA-STAD (Data Release 31.0) cohort database. The transcriptomic and clinical data of 431 GC samples and 45 GC patients treated with pembrolizumab monotherapy were collected from the GSE84437 and the PRJEB25780, respectively. The expression matrix of tumor and normal tissues from the GSE54129 cohort (111 GC samples and 21 normal controls) and the GSE13861 cohort (66 GC samples and 19 normal controls), respectively. The single-cell RNA sequencing data of 13 fresh human tissue samples from nine GC patients were retrieved from the GSE134520 dataset. The process and specific cohorts used in the analysis were depicted in Figure 5.

### Analysis of senescence-related DEGs and functional enrichment

The gene expression profiles were analyzed in tumorous and normal samples of the TCGA-STAD cohort via the “limma” R package (Version 3.28.14), and genes with a false discovery rate (FDR) <0.05 were selected as DEGs. Gene ontology (GO) and KEGG analysis were conducted for functional enrichment of the DEGs utilizing the “clusterProfiler” R package (Version 3.0.4).

### Model development and verification

The prognostic value of every single senescence-related gene was examined by univariable cox regression using the R package “survival” according to the log_2_ (Fragments Per Kilobase Million + 1) value of each gene. The LASSO method (the “glmnet” R package, Version 4.3) was utilized for model construction in the training set (the TCGA-STAD cohort) based on the DEGs with a significant association with OS [40]. The number of genes input for model construction was selected according to the minimum penalty parameter (λ) by ten-fold cross-validation. The risk-score was determined as follows:


$$Risk\;score=\sum_{i=1}^{n}expi\times \beta i$$

“*n*” depicted the number of genes involved in the model, while“expi” and “β*i*” represents the mRNA level and regression coefficient of gene *i*, respectively.

Assigned with a risk-score, patients were stratified into high- or low-risk groups by the median value in the cohort. The R package “survival” (version 3.4.0) was applied for survival analysis comparing the OS between the high- and low-risk groups. The prognostic value of the model was evaluated with the AUC and C-index values, and visualized by the receiver operating characteristic (ROC) curve by the “timeROC” R package (Version 0.4). The GSE84437 and the GSE13861 cohorts were utilized for validation.

### Tumor immune infiltration analysis

Based on the cell types categorized by the deconvolution approach in CIBERSORT [41], the density of immune cells in tumor was identified. [42]. The potential association of the risk-score and TIME was analyzed by Spearman correlation.

### Association analysis of the risk-score and drug sensitivities

We analyzed the response to pembrolizumab monotherapy in GC patients from the PRJEB25780 cohort. Based on the genomics of drug sensitivities in cancer (GDSC) database (https://www.cancerrxgene.org), we calculated the correlations (Spearman correlation analysis) of the half maximal inhibitory concentration (IC50) with the mRNA expression and the risk-score. The results are obtained by the “pRRophetic” (Version 4.0.2) and the “ggplot2” (Version 3.3.6) R packages. *P* values were adjusted by the FDR method.

### Expression verification and localization of the senescence-related genes

The mRNA levels of the 6 genes involved in the signature were compared between GC tumors and normal tissues from two more cohorts (GSE54129, 111 GC and 21 controls; GSE13861, 66 GC and 19 controls), together with the expression levels compared among different cell types in GC tissues from the GSE134520 dataset, with annotation from the Tumor Immune Single-cell Hub (TISCH) database for cell identification. IHC staining figures in GC tissues and normal gastric samples were obtained from The Human Protein Atlas database (THPA, https://www.proteinatlas.org/).

### Statistical analysis

Statistical results generated in this study were conducted in R (Version 3.6.0), SPSS (Version 23.0), and GraphPad Prism (Version 8). Wilcox test was used to analyze the association between the senescence-related gene signature and immune characteristics. Survival analyses were conducted by the Log-rank test, with visualization by the Kaplan-Meier (KM) curves. The independence of the prognostic signature was verified by univariable and multivariable Cox regression, with the input of significant variables into the multivariable analysis by *P* < 0.05. The accuracy of the signature was examined and depicted by the area under the curve (AUC). If not stated above, *P* < 0.05 illustrated statistical significance.

## Supplementary Materials

Supplementary Figure 1

Supplementary Tables
