# Supplementary material for: Senescence-related genes define prognosis, immune contexture, and pharmacological response in gastric cancer
Source: Aging (Albany NY). 2023 Feb 16;15(8):2891–905. doi: 10.18632/aging.204524 (PMC10188345; doi:10.18632/aging.204524)
Supplement: Supplementary Figure 1 [file aging-15-204524-s001.pdf]

SUPPLEMENTARY FIGURE

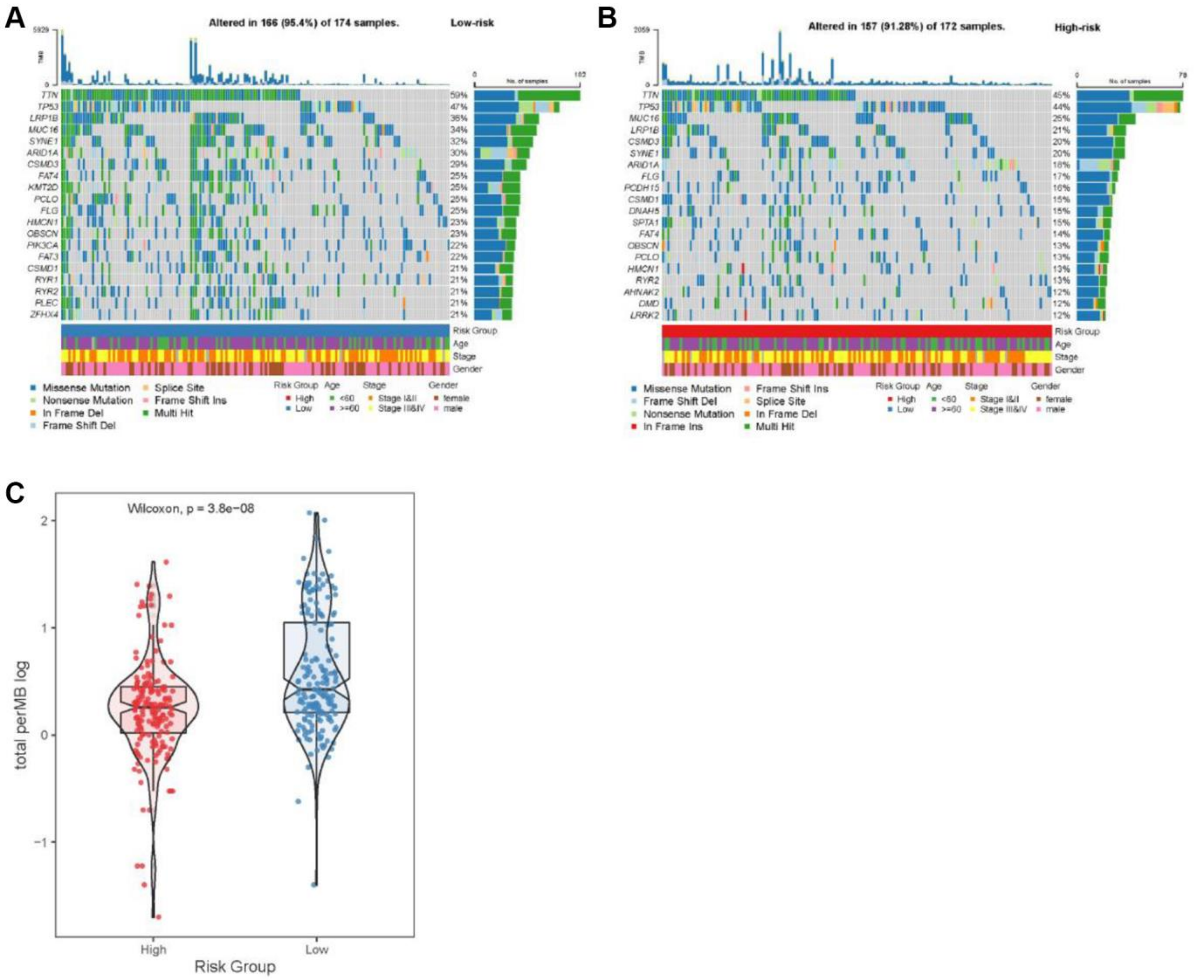

Supplementary Figure 1. Somatic variants and TMB in the TCGA cohort. (**A**) Oncoprint plot displaying the top 20 frequently mutated genes in low-risk groups. (**B**) Oncoprint plot displaying the top 20 frequently mutated genes in high-risk groups. (**C**) Somatic TMB between high- and low-risk groups in the TCGA cohort. Abbreviation: TMB: tumor mutational burden.
