# Supplementary material for: Senescence-related genes define prognosis, immune contexture, and pharmacological response in gastric cancer
Source: Aging (Albany NY). 2023 Feb 16;15(8):2891–905. doi: 10.18632/aging.204524 (PMC10188345; doi:10.18632/aging.204524)
Supplement: Supplementary Tables [file aging-15-204524-s002.pdf]

SUPPLEMENTARY TABLES

Supplementary Table 1. Clinical cohorts analyzed in this study.

	TCGA-STAD	GSE84437	GSE13861
**No. of patients**	348	431	64
**Sex**			
Female	123 (35.34%)	137 (31.79%)	19 (29.69%)
Male	225 (64.66%)	294 (68.21%)	45 (70.31%)
**Age**			
<65	149 (42.82%)	267 (61.95%)	39 (60.94%)
≥65	196 (56.32%)	164 (38.05%)	25 (39.06%)
**Stage**			
I	46 (13.22%)	31 (16.32%)	12 (18.75%)
II	110 (31.61%)	29 (15.26%)	11 (17.19%)
III	144 (41.38%)	71 (37.37%)	25 (39.06%)
IV	34 (9.77%)	59 (31.05%)	16 (25.00%)
**Molecular classification**			
EBV	26 (7.47%)	Not available	Not available
CIN	180 (51.72%)	Not available	Not available
MSI	57 (16.38%)	Not available	Not available
GS	63 (18.1%)	Not available	Not available
***H. pylori* infection**			
Negative	139 (39.94%)	Not available	Not available
Positive	18 (5.17%)	Not available	Not available
**With radiation therapy**			
No	229 (65.8%)	Not available	Not available
Yes	45 (12.93%)	Not available	Not available

Abbreviations: EBV: Epstein-Barr virus; CIN: chromosomal instability; MSI: microsatellite instability; GS: genomicstability.

Supplementary Table 2. The six DEGs used in the risk-score model.

The six DEGs used in the risk-score model		
**Signature**	**Description**	**Entrez gene summary**
SERPINE1	Serpin peptidase inhibitor, clade E (nexin,plasminogen activator inhibitor type 1), member 1	*SERPINE1* encodes a member of the serine proteinase inhibitor (serpin) superfamily, whichis the principal inhibitor of tissue plasminogen activator (tPA) and urokinase (uPA).
FEN1	Flap structure-specific endonuclease 1	*FEN1* is a major component of the base excision repair pathway for DNA repair systems and plays important roles in maintaining genomic stability through DNA replication and repair.
PDGFRB	Platelet-derived growth factor receptor,beta polypeptide	*PDGFRB* binds to platelet-derived growth factor (PDGF)-B and -D, and is highly expressedin fibroblasts, pericytes and other cells of mesenchymal origin.
SNCG	Synuclein, gamma (breast cancer-specificprotein 1)	*SNCG* is a chaperone protein and exists mainly in the cytoplasm. SNCG confers chemoresistance, and is a potential unfavorable biomarker for multiple types of cancer.
TCF3	Transcription factor 3	*TCF3* (also known as Tcf7l1) belongs to the Lef/Tcf family of transcription factors, all of which contain a highly conserved HMG domain that binds to a conserved recognition sequence, as well as domains that interact with the transcriptional activator β-catenin and Groucho/TLE-family corepressors.
APOC3	Apolipoprotein C-III	*APOC3* is a potential prognostic biomarker for several types of malignant tumors, which is reported for having an association with the lipoprotein-associated phospholipase A2, catalyzing the hydrolysis of oxidized low-density lipoprotein, together with releasing inflammatory products and being found in the ruptured plaques of human atherosclerotic lesions.
